# Unraveling the impact of laser refractive surgery on corneal ectasia: an *in silico* study

**DOI:** 10.3389/fbioe.2025.1548539

**Published:** 2025-02-26

**Authors:** Benedetta Fantaci, José Félix Rodriguez Matas, Vittoria Squartecchia, Lucia Vavassori, Begoña Calvo

**Affiliations:** ^1^ Aragon Institute of Research Engineering (I3A), Universidad de Zaragoza, Zaragoza, Spain; ^2^ LaBS, Department of Chemistry, Materials and Chemical Engineering “Giulio Natta”, Politecnico di Milano, Milan, Italy; ^3^ Bioengineering, Biomaterials and Nanomedicine Networking Biomedical Research Centre (CIBER-BBN), Universidad de Zaragoza, Zaragoza, Spain

**Keywords:** corneal biomechanics, laser refractive surgery, PRK, LASIK, SMILE, ectasia, finite element model, *in silico* analysis

## Abstract

**Introduction:**

Laser refractive surgeries are a safe option for low-to-moderate refractive corrections, providing excellent visual outcomes. Over the years, various procedures have been introduced into clinical practice, but the most performed today remain Photorefractive Keratectomy (PRK), Laser *In-Situ* Keratomileusis (LASIK), and Small Incision Lenticule Extraction (SMILE). Although laser refractive treatments are considered safe, clinicians have focused on the risk of post-surgical ectasia, a rare but serious complication. Ectasia is characterized by progressive corneal thinning and steepening, leading to vision distortion, irregular astigmatism, and in some cases, a reduction of visual acuity. It is still debated whether laser refractive surgeries can cause ectasia as an iatrogenic condition or merely accelerate the progression of an underlying corneal pathology, not detected during pre-surgical screening. The proposed work investigates the relationship among three laser refractive surgeries (PRK, LASIK and SMILE), currently performed in clinical practice, and ectasia onset and progression by means of an *in silico* analysis.

**Methods:**

An average 3D finite-element corneal model is developed and a pathological area, characterized by reduced stiffness of varying severity grades, is defined to analyze its influence on ectasia development and progression in the pre-surgical state. Three laser treatments (PRK, LASIK and SMILE) are simulated on healthy and pathological models. Pre- and post-surgical conditions are compared to check whether any procedure worsens the pre-surgical pathological state. The optomechanical effect of each procedure on the cornea is analyzed at both healthy and pathological conditions and compared to establish which refractive procedure mostly affects corneal structure.

**Results and discussion:**

While the three refractive procedures showed different behaviors in terms of mechanical changes affecting the cornea, from an optical perspective, as the pathology severity worsened, none of the surgeries caused a worsening in the cone’s severity with respect to pre-surgical pathological conditions. This result suggests that surgeries may have a limited role in causing post-surgical ectasia, as it seems more plausible that they accelerate the progression of an underlying pathological condition. Among the three procedures, PRK was found to be the least invasive treatment from a mechanical perspective, while SMILE showed the greatest impact on the posterior surface, suggesting a potential long-term risk for ectasia progression.

## 1 Introduction

Laser refractive surgeries are a safe and effective option for low-to-moderate refractive corrections, providing excellent visual outcomes and allowing to achieve spectacle independence (Kim et al., 2019). As the outermost layer of the eye, the cornea is responsible for two-thirds of its refractive power and is easily accessible for receiving such treatments. Over the past 40 years, various procedures have been introduced into clinical practice, all based on the same principle: reshaping the anterior surface of the cornea with a laser to correct refractive defects (myopia, astigmatism, or hyperopia) by shifting the focal point onto the retina.

Photorefractive Keratectomy (PRK) was the first procedure to be invented in the 1980s, involving surface ablation of the stroma with an excimer laser after removing the epithelium. After the treatment, the epithelium regenerates over the ablated stroma. PRK is known to preserve corneal biomechanics better than other procedures, as it leaves a higher percentage of residual stromal bed (RSB), which helps support the cornea under the remaining post-surgical loads. However, in this procedure, the Bowman’s layer–a thin, non-regenerating layer between the epithelium and stroma that helps maintain corneal shape–is removed during laser ablation. Consequently, the removal of both the epithelium and Bowman’s layer triggers a wound healing process that can impact refractive correction and lead to scarring, causing post-surgical discomfort for the patient (Fogla et al., 2020).

Later, in the 1990s Laser *In Situ* Keratomileusis (LASIK) surpassed PRK, establishing itself as the preferred procedure by the surgeons. In LASIK a hinged flap is created with a femto-second laser and lifted, the tissue below is ablated with an excimer laser and the flap is put back in place after the ablation, with no sutures needed. The creation of a flap promotes faster recovery and less discomfort by preserving the epithelium, but results in a lower percentage of RSB, thereby reducing corneal biomechanical strength (Sutton et al., 2014; Kim et al., 2019).

Finally, the most recent procedure to enter the market in the 2000s was Small Incision Lenticule Extraction (SMILE), where a lenticule, tailored for the desired refractive target, is created with the femto-second laser in the corneal stroma and extracted through a small incision. SMILE is a flapless procedure, believed to better preserve corneal structural integrity (Reinstein et al., 2014).

Although laser refractive treatments are generally considered safe with few adverse effects, clinicians and scientists have focused on the risk of post-surgical ectasia, a rare but serious complication (Wolle et al., 2016). The incidence variability of ectasia after LASIK reported in the literature ranges from 0.013% (13 eyes per 100,000) to 0.935% (935 eyes per 100,000), while its occurrence after PRK and SMILE remains poorly understood (Moshirfar et al., 2021). Ectasia is characterized by progressive corneal thinning and steepening, leading to vision distortion, irregular astigmatism, and in some cases, a significant loss of visual acuity. While it can develop within months after surgery, it may also appear years later (Wolle et al., 2016). It usually first manifests in one eye as a bulge in the infero-temporal region of the cornea and later in time in the fellow eye (Santodomingo-Rubido et al., 2022).

The development and progression of ectasia are believed to be closely related to the health of the corneal tissue (Santhiago et al., 2014), particularly the stroma. The stroma is primarily responsible for the cornea’s biomechanical response, accounting for 90% of its total thickness. It features a highly organized structure of collagen fibers arranged into fibrils, which are grouped into lamellae and embedded within the extracellular matrix (ECM). The ECM plays an adhesive role, maintaining the lamellae’s arrangement. In ectasia, this organization is disrupted due to ECM alterations that reduce matrix stiffness and impair its embedding function, allowing the fibers to move freely and eventually break (Santodomingo-Rubido et al., 2022). As the tissue degrades, the cornea thins and bulges outward under the influence of intraocular pressure (IOP).

Ectasia histopathology has been extensively described in the literature, but its etiology remains a topic of debate, as the exact triggering cause is still unclear. Environmental factors, such as chronic eye rubbing and contact lens wear Ferrari and Rama (2020), may contribute to its progression, although genetic predisposition is considered the primary factor in the disease’s genesis Santodomingo-Rubido et al. (2022).

The role of refractive surgeries in triggering ectasia remains unclear. It is still debated whether these procedures cause ectasia as an iatrogenic condition or merely accelerate the progression of an underlying corneal pathology (Moshirfar et al., 2021), not detected during pre-surgical screening. Several risk factors have been identified as potential contributors to the disease and are assessed during pre-surgical evaluation to determine patient eligibility for laser refractive treatment. Pre-existing corneal abnormalities significantly increase the risk of post-surgical ectasia (Martínez-Abad and Piñero, 2017; Santhiago et al., 2016). Even minor geometric irregularities may indicate the presence of an underlying condition. For example, high myopia or astigmatism require more invasive refractive corrections, which can lead to structural instability due to the ablation of a thicker portion of the corneal tissue. Each surgical procedure inevitably weakens corneal biomechanical strength to varying degrees, primarily due to the reduction in corneal thickness. Thin corneas are more prone to post-surgical complications (Santhiago et al., 2014; Wolle et al., 2016). Moreover, depending on the chosen procedure, the reduction in thickness may be greater, as the flap or cap thickness also contributes to a lower percentage of RSB. As a result, surgeons typically avoid performing surgery when the RSB is less than 250 µm (Randleman et al., 2003). In general, preserving the RSB is crucial for maintaining corneal biomechanical strength.

Given the lack of a clear understanding of ectasia development and behavior, various authors have attempted to gain a deeper understanding of the disease using finite element (FE) models. These models are versatile tools that enable the investigation of multiple aspects of the disease, from structural to optomechanical changes, and allow to analyze the influence of different corneal tissue components.

Many studies focused on reproducing the progression of the disease in terms of corneal bulging and thinning, with the aim to better understand the underlying processes that may trigger the development of the pathology (Pandolfi and Manganiello, 2006; Gefen et al., 2009; Falgayrettes et al., 2023). Other authors exploited FE models of ectatic corneas to investigate the optomechanical effect of treatments currently applied in clinics to stop the progression, such as cross-linking (Roy and Dupps, 2011; Roy et al., 2013) or intracorneal segment ring implantation (Flecha-Lescún et al., 2018; Ariza-Gracia et al., 2020). The work by Dupps et al. (Dupps and Seven, 2016) investigated the risk of developing ectasia after LASIK, by proposing strain as a metric for the expression of structural risks in laser refractive surgeries. The same research group highlighted the advantages of employing patient-specific computational analyses in the mechanical evaluation of corneal structure after laser refractive surgery (Vahdati et al., 2016). Recently, FE models have been employed to predict post-surgical ectasia in patients who underwent refractive treatments and developed the condition (Francis et al., 2023).

The proposed work fits within the finite element modeling framework and primarily investigates the role of laser refractive surgeries in contributing to post-surgical ectasia through FE analysis, aiming to determine which refractive treatment is the most invasive from a biomechanical perspective, if any. Corneal FE models, characterized by a weak area of increasing severity, will be analyzed at pre- and post-surgical conditions. Simulations of the three refractive procedures (PRK, LASIK, and SMILE) will be conducted on pathological corneas to analyze their optomechanical impact on ectasia and assess whether any procedure exacerbates the pre-existing pathological condition, highlighting its potential role in ectasia progression. Both the optical and mechanical effects of each procedure on the cornea will be examined under healthy and pathological conditions. The results will then be compared to determine which procedure has the greatest impact on corneal biomechanics, which is the least mechanically invasive, or whether their optomechanical effects are comparable.

## 2 Materials and methods

In the following section, we will describe the model’s characteristics and the set-up of the laser refractive surgeries simulations for both healthy and pathological corneas, characterized by an area of reduced stiffness.

### 2.1 Material model

**FIGURE 1:**
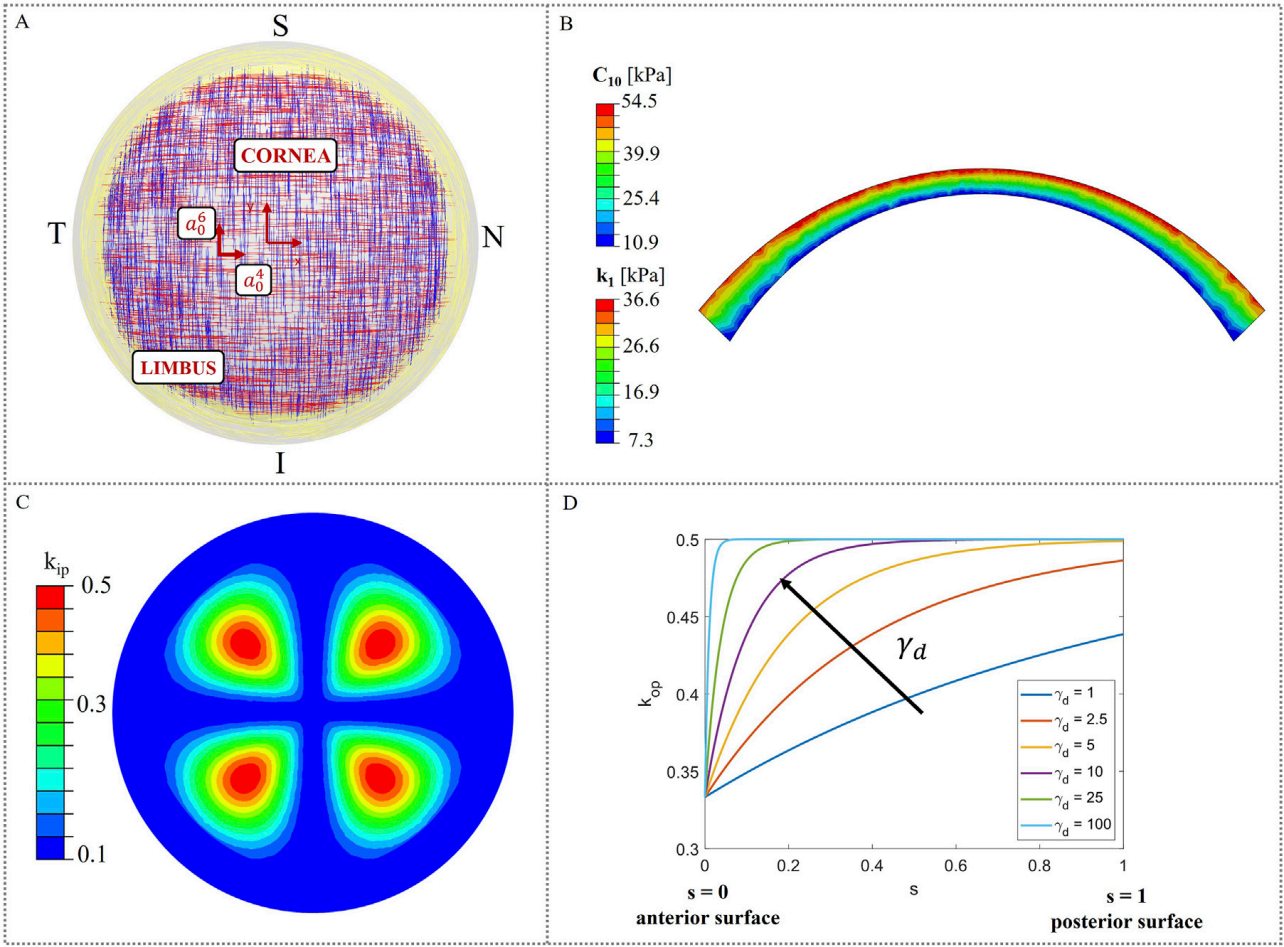
Material model characteristics. **(A)** Theoretical distribution of collagen fibers (Meek and Boote, 2009); **(B)** Heterogeneous distribution of material properties (C_10_ and k_1_) in corneal thickness; **(C)** In-plane dispersion k_ip_ distribution in the corneal model; **(D)** Variation of the out-of-plane dispersion k_op_ as a function of the local coordinate s.

Corneal tissue is made of five layers (epithelium, Bowman’s layer, stroma, Descemet’s membrane and endothelium), among which the stroma is responsible for the biomechanical response, since it constitutes 90% of the corneal thickness. The stroma is arranged in a highly organized structure, contributing to the transparency that characterizes the cornea as a lens (Sridhar, 2018). In the posterior third of the stroma, the lamellae are orthogonally oriented along the nasal-temporal and inferior-superior directions, while in the anterior two-thirds, they progressively adopt an isotropic distribution. Toward the limbus, the lamellae assume a circumferential orientation (Winkler et al., 2011) (Figure 1A).

In this work, the cornea was modeled as a monolayer stromal tissue, following the assumption made in our previous study (Fantaci et al., 2024a). This approach is widely adopted in literature (Pandolfi et al., 2009; Sánchez et al., 2014; Simonini and Pandolfi, 2015; Simonini et al., 2022), given the stroma’s primary role in determining the corneal mechanical response and the current lack of mechanical characterization for the other corneal layers.

Figure 1A illustrates a theoretical scheme of fibers orientation; however, collagen fibers exhibit varying degrees of dispersion depending on their location within the cornea and are not perfectly aligned. As a consequence, in-plane and out-of-plane dispersion (Figures 1C, D), as derived by (Pandolfi and Holzapfel, 2008; Wang and Hatami-Marbini, 2021), must be considered to account for point-wise fibers distribution.

The following strain energy density function (SEDF) for a nearly-incompressible material was used to model corneal tissue behavior (Equation 1, Wang and Hatami-Marbini, 2021):

$$\psi \left(\bar{C},J\right)=\psi^{\mathrm{matrix}}\left({\bar{I}}_{1},{\bar{I}}_{2}\right)+\underset{i=4,6}{\Sigma }\psi^{\mathrm{fibers}}\left(\bar{C},H_{i}\right)+\psi \left(J\right),$$ (1)

where $\psi^{\mathrm{matrix}}\left({\bar{I}}_{1},{\bar{I}}_{2}\right)$ represents the isotropic contribution of the ECM, $\psi^{\mathrm{fibers}}\left(\bar{C},H_{i}\right)$ accounts for the anisotropic contribution given by the fibers of the model, and ψ(J) is the volumetric term.

$\bar{C}$ is the deviatoric right Cauchy-Green tensor (Equation 2):

$$\bar{C}=J^{-2/3}C,$$ (2)

where $C=F^{T}F$ is the right Cauchy-Green tensor. The invariants of $\bar{C}$ are (Equations 3–5):

$${\bar{I}}_{1}=tr\bar{C},$$ (3)

$${\bar{I}}_{2}=\frac{1}{2}\left({\bar{I}}_{1}^{2}-\bar{C}:\bar{C}\right),$$ (4)

$${\bar{I}}_{3}=\det\left(\bar{C}\right)=1.$$ (5)

The anisotropy of the cornea is characterized by the fabric tensor $H_{i}$ , i={4,6} , that accounts for fibers dispersion and is defined as (Equation 6):

$$H_{i}=A1+Ba_{0}^{i}⊗a_{0}^{i}+\left(1-3A-B\right)a_{n}⊗a_{n},$$ (6)

with constants $A=2k_{ip}k_{op}$ and $B=2k_{op}\left(1-2k_{ip}\right)$ . The unit vectors $a_{0}^{4}$ and $a_{0}^{6}$ are associated with the mean preferential directions of the two families of fibers that characterize the corneal stroma (see Figure 1A), whereas $a_{n}$ is the unit vector normal to the cornea, that identifies the out-of-plane direction.

The following equations for in-plane dispersion k_ip_ were used (Equations 7, 8, Figure 1C):

$$k_{ip}\left(\theta \right)=\left(\frac{k_{ip}^{\min}+k_{ip}^{\max}}{2}\right)-\left(\frac{k_{ip}^{\max}-k_{ip}^{\min}}{2}\right)\cos4\theta$$ (7)

$$k_{ip}\left(\theta ,r\right)=k_{ip}^{\min}+\frac{1}{2}\left(k_{ip}\left(\theta \right)-k_{ip}^{\min}\right)\left(1-\cos\frac{2\pi r}{R_{TZ}}\right),$$ (8)

where $k_{ip}^{\min}=0.1$ is the minimum value (completely anisotropic distribution), $k_{ip}^{\max}=0.5$ is the maximum (completely isotropic distribution) and $R_{TZ}=5.5$ mm is the radius of the transition zone from the cornea to the limbus.

Out-of-plane dispersion varies in depth, being the fibers more orthogonally distributed within the posterior two-thirds and more isotropically oriented within the anterior third. To define $k_{op}$ , a local coordinate s∈[0,1] must be used, parallel to the normal unit vector. This local coordinate is 0 at the anterior surface and one at the posterior surface (Figure 1D). The following equation for the out-of-plane dispersion was used (Equation 9):

$$k_{op}\left(s\right)=k_{op}^{\min}+\left(k_{op}^{\max}-k_{op}^{\min}\right)\left(1-e^{-\gamma_{d}s}\right),$$ (9)

where $k_{op}^{\min}=1/3$ (completely isotropic distribution) and $k_{op}^{\max}=1/2$ (completely anisotropic distribution) and the constant $\gamma_{d}$ controls the non linearity of Equation 9 (Figure 1D). A value of $\gamma_{d}=1$ was assigned to the model.

To describe the behavior of the ECM, the Neo-Hookean model was used (Equation 10):

$$\psi^{\mathrm{matrix}}\left({\bar{I}}_{1}\right)=C_{10}\left({\bar{I}}_{1}-3\right),$$ (10)

where C_10_ regulates matrix’s stiffness.

The hyperelastic Holzapfel-Gasser-Odgen model with dispersion parameters was used (Pandolfi and Holzapfel, 2008; Wang and Hatami-Marbini, 2021) to model the anisotropic contribution of the collagen fibers (Equation 11):

$$\psi^{\mathrm{fibers}}\left(\bar{C},H_{i}\right)=\underset{i=4,6}{\sum }\frac{k_{1}}{2k_{2}}\left(e^{k_{2}{\left({\bar{I}}_{i}^{*}-1\right)}^{2}}-1\right),$$ (11)

where k_1_ and k_2_ are constants that control the fibers stiffness and fibers non-linearity, respectively. The distorsional generalized invariant ${\bar{I}}_{i}^{*}$ has the following form (Equations 12–14):

$${\bar{I}}_{i}^{*}=H_{i}:\bar{C}=2k_{ip}k_{op}{\bar{I}}_{1}+2k_{op}\left(1-2k_{ip}\right){\bar{I}}_{i}+\left(1-6k_{ip}k_{op}-2k_{op}\left(1-2k_{ip}\right)\right){\bar{I}}_{n},$$ (12)

with

$${\bar{I}}_{i}=\bar{C}:a_{0}^{i}⊗a_{0}^{i},$$ (13)

$${\bar{I}}_{n}=\bar{C}:a_{n}⊗a_{n}.$$ (14)

Finally, the volumetric term is (Equation 15):

$$\psi \left(J\right)=\frac{1}{D}{\left(\log J\right)}^{2},$$ (15)

where $D=3.6\cdot 10^{-4}$ is the volumetric constant.

Stromal tissue is characterized by a stiffness gradient from the posterior to the anterior surface, throughout its thickness, as demonstrated by (Randleman et al., 2008). In this work, depth-dependent stiffness was introduced in the model, by assigning heterogeneous material properties to the model. To reproduce the stiffness gradient characteristic of human corneas, the corneal model was divided into four different layers (Figure 1B) and material properties with a linear variation from the posterior surface to the anterior were assigned (Randleman et al., 2008; Reinstein et al., 2013; Dias and Ziebarth, 2013; Dupps and Seven, 2016). More specifically, the maximum values of the material properties were assigned at the anterior surface and gradually decrease until they reach a 20% of the initial value at the posterior surface (Falgayrettes et al., 2023). We assumed a linear variation for the matrix stiffness constant $C_{10}$ and for the fibers stiffness constant $k_{1}$ , while the constant that controls fibers non-linearity $k_{2}$ was kept constant throughout the entire thickness (Pandolfi and Holzapfel, 2008). The constant values assigned to each layer were determined through an optimization process aimed at matching the apical displacement of a homogeneous monolayer corneal model reported in the literature (Wang and Hatami-Marbini, 2021). The material parameters assigned to each layer of our model are shown in Table 1.

**TABLE 1:** Corneal material parameters along the thickness.

	C_10_ [kPa]	k_1_ [kPa]	k_2_ [-]	k_ip_-k_op_ [-]
First layer (anterior surface)	54.5	36.3	400	Equations 8, 9
Second layer	39.9	26.6	400	Equations 8, 9
Third layer	25.4	16.9	400	Equations 8, 9
Fourth layer (posterior surface)	10.9	7.3	400	Equations 8, 9

### 2.2 Likelihood of ectasia onset: local stiffness reduction

**FIGURE 2:**
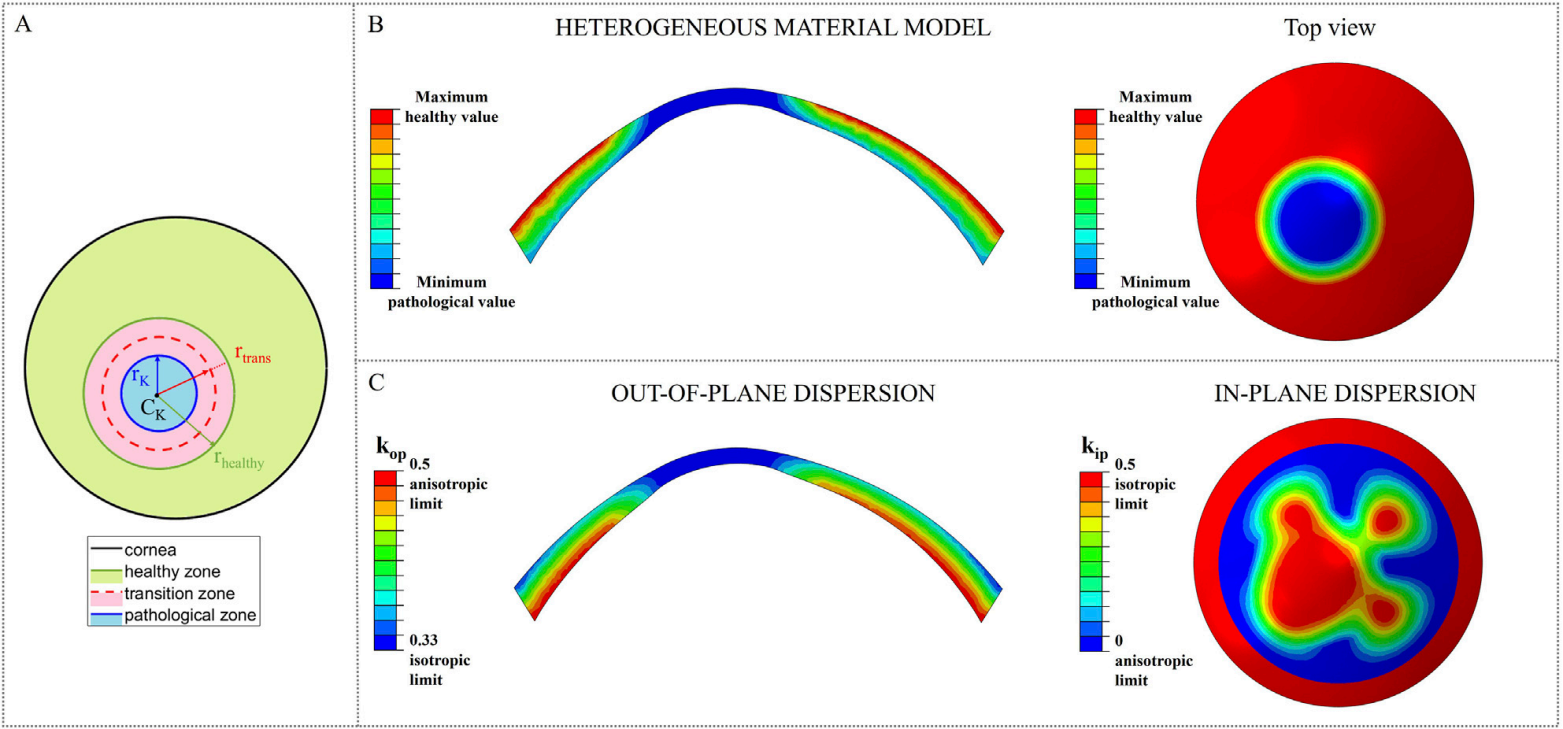
Stiffness reduction of the material properties of the model. **(A)** Location and dimension of the pathological area; **(B)** Pathological material properties distribution in the heterogeneous material models for constants C_10_ and k_1_; **(C)** Change of k_ip_ and k_op_ towards their isotropic limit due to the loss of fibers orientation in the pathological area.

To analyze the likelihood of ectasia onset and progression in pre- and post-surgical conditions, a circular weakened area of radius r_K_ = 1.5 mm and center C_K_ = [-0.663, −1.019] [mm] (Eliasy et al., 2020), characterized by reduced stiffness, was introduced in the nasal inferior position, where ectasia usually develops (Figure 2A). Around the central pathological area of radius r_K_, a transition zone of radius r_trans_ was defined to avoid any abrupt change of properties from the pathological to the healthy tissue. Ten different cases with increasing pathology severity were considered (Table 2); each case represents a different degree of severity correlated to a specific stiffness reduction γ in the pathological area. Starting from healthy corneal tissue properties (labeled as γ = 1), a 10% reduction of the material properties stiffness is progressively applied to the pathological area until reaching a maximum stiffness reduction of 90% with respect to the initial healthy properties (labeled as γ = 0.1) (i.e., γ decreases from 1 to 0.1 with step 0.1). These stiffness reductions were applied to the ECM stiffness constant $C_{10}$ and to the fibers stiffness constant $k_{1}$ , while the fibers non-linearity constant $k_{2}$ was left unvaried (Figure 2B). The dispersion parameters $k_{ip}$ and $k_{op}$ were linearly changed until reaching their isotropic limits, 0.5 and 0.33, respectively, given the disorganization of collagen fibers, typically observed in keratoconic corneas (Alkanaan et al., 2017) (Figure 2C). The stiffness reduction was applied to each layer of the model for all the cases considered (Figure 2B; Table 2).

**TABLE 2:** Material properties in the pathological area for the ten selected cases with increasing severity corresponding to a stiffness reduction γ.

Layer	γ	1	0.9	0.8	0.7	0.6	0.5	0.4	0.3	0.2	0.1
1	C_10_ [kPa]	54.5	49.1	43.6	38.2	32.7	27.3	21.8	16.4	10.9	5.5
	k_1_ [kPa]	36.3	32.7	29	25.4	21.8	18.2	14.5	10.9	7.3	3.6
2	C_10_ [kPa]	39.9	35.9	31.9	27.9	23.9	20	16	12	8	4
	k_1_ [kPa]	26.6	23.9	21.3	18.6	16	13.3	10.6	8	5.3	2.7
3	C_10_ [kPa]	25.5	23	20.4	17.9	15.3	12.8	10.2	7.7	5.1	2.6
	k_1_ [kPa]	16.9	15.2	13.5	11.8	10.1	8.5	6.8	5.1	3.4	1.7
4	C_10_ [kPa]	10.9	9.8	8.7	7.6	6.5	5.5	4.4	3.3	2.2	1.1
	k_1_ [kPa]	7.3	6.6	5.8	5.1	4.4	3.7	2.9	2.2	1.5	0.7
Transition zone Width	[mm]	0	0.167	0.33	0.5	0.67	0.83	1	1.167	1.33	1.5

As the degree of pathology severity increases, the transition zone expands: starting from the less severe case (γ = 0.9), a transition zone of 0.1667 mm was defined and gradually expands throughout the pathological cases, until reaching a final width of 1.5 mm (r_trans_ = r_healthy_ = 3 mm) for the most severe case (γ = 0.1) (Table 2). A linear variation of the material properties from the pathological to the healthy value was assigned to the transition zone of the model, to smooth the stiffness change between the two areas of the cornea.

### 2.3 Laser refractive surgeries simulations

A 3D FE conic model (Fantaci et al., 2024a) with average dimensions (anterior surface: apical radius R_AS_ = 8.18 mm and asphericity Q_AS_ = −0.25; posterior surface: apical radius R_PS_ = 6.63 mm and asphericity Q_PS_ = −0.26; central corneal thickness: CCT = 579 μm; corneal diameter \diameter = 12 mm) was built to simulate the laser refractive surgeries. The FE model was meshed with quadratic tetrahedrons of 0.1 mm dimension with the software ANSA version 22.0.1 by BETA CAE Systems. Mesh sensitivity analysis was conducted in a previous study (Fantaci et al., 2024a). The limbus was fixed to ensure model’s stability and an average physiological IOP of 15 mmHg was applied to the posterior surface of the cornea to mimic the pressure acting inside the eye cavity. The zero-pressure algorithm from (Ariza-Gracia et al., 2016) was used to recover the unpressurized geometry, that became the initial geometry for the surgeries simulation. The stress-free configuration was recovered only in the healthy case, and then the pathological area was incorporated. All the simulations were run with the proprietary software ABAQUS 2022.

**FIGURE 3:**
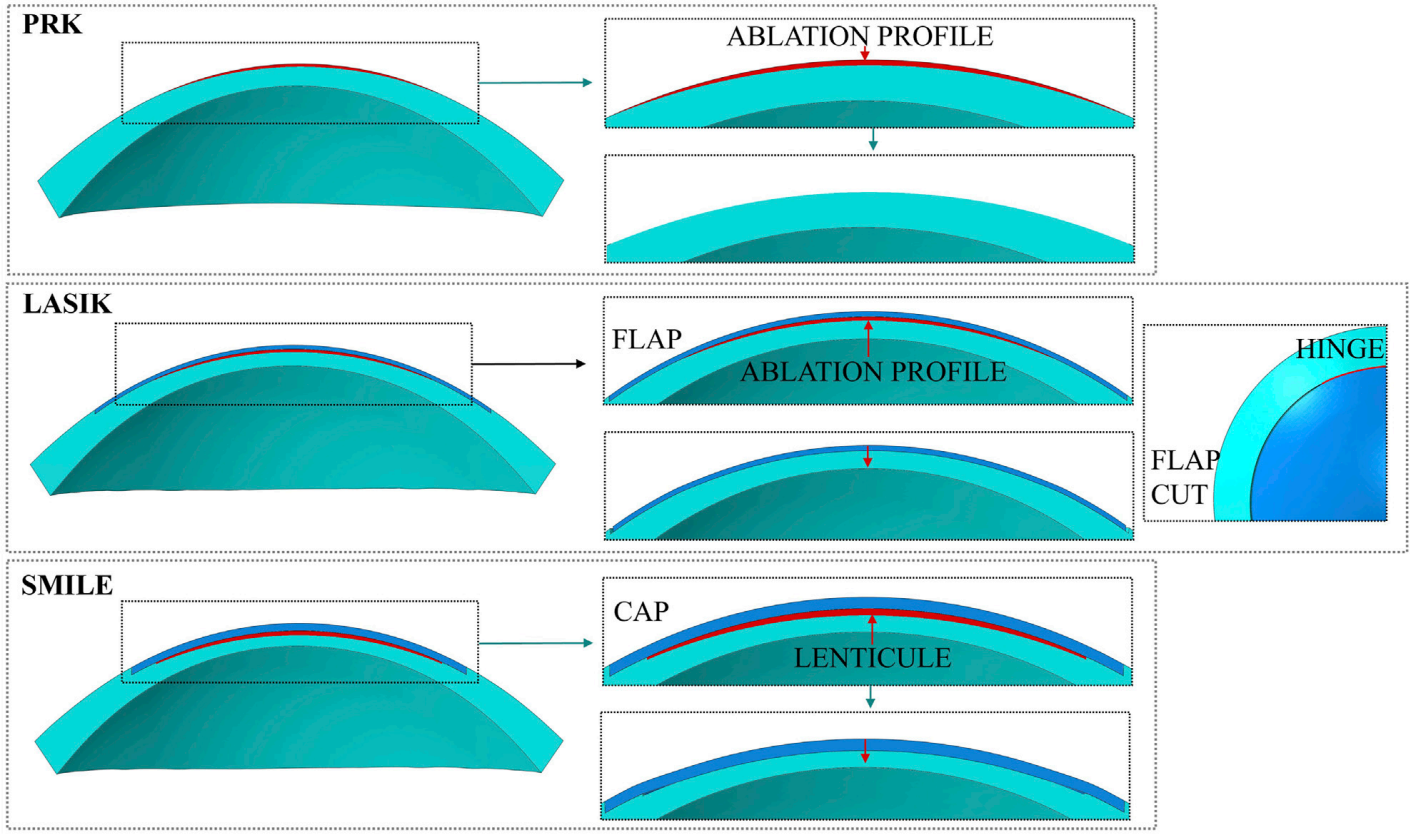
FE models of the laser refractive surgeries.

For each surgery simulation (shown in Figure 3), the ablation profile for −4 D myopic correction was defined by following Equations 1, 5, 6 from our previous work (Fantaci et al., 2024a) and the ablation depth was established by following Table 2 of the same work. In all the surgical simulations, an optical zone of \diameter = 6.5 mm was defined, that represents the diameter of the ablation tissue that will be removed. This value is the most used in clinical practice for the three procedures and rarely varies.

When constructing the finite element model, specific adjustments must be made depending on the selected surgery procedure.

#### 2.3.1 PRK

PRK model is built so that two different element sets are created to separate the ablation volume from the rest of the cornea (i.e., the post-surgical corneal volume). The ablation depth was 60 µm for simulating a myopic correction of −4 D in PRK, leaving a RSB of 519 µm.

PRK surgery consisted of two steps (Figure 3):

• first step - Pressurization: the model is pressurized with an IOP applied to the posterior surface of the cornea.
• second step - Ablation: the ablation volume is removed.

#### 2.3.2 LASIK

In LASIK treatment, a hinged corneal flap is created with a femtosecond laser and lifted to allow direct ablation of the stroma using an excimer laser. The characteristic dimension of the flap selected to simulate the surgery were the following (Moshirfar et al., 2019): flap \diameter = 9 mm, flap thickness = 100 μm, hinge length = 4 mm. The hinge of the flap is always placed in the superior central region of the cornea, to reduce the probability of post-surgical flap dislocation. As for PRK, LASIK surgery required an ablation depth of 60 µm. The RSB, considering flap and lenticule thickness, was of 419 µm.

Before running the simulation, contacts between the parts of the corneal geometry must be defined and adjusted during the simulation. Specifically, a hard contact with infinite friction is established between the bottom surface of the flap and the underlying corneal surface to prevent penetration and sliding. This contact ensures model continuity as an initial condition when the model is pressurized. A hard contact with friction coefficient of 0.5 is then defined between the flap and the post-surgical cornea, activated in the second step when the surgery is simulated. The decision to restrict the flap from freely sliding on the underlying cornea–despite this being its typical behavior during surgery–was made because the focus of this study is on analyzing the effects of laser surgery on ectasia onset after the corneal geometry has stabilized (Katzengold et al., 2021; Wang et al., 2022), rather than in the immediate post-surgical phase.

LASIK surgery consisted of three steps (Figure 3):

• First step - Pressurization: the model is pressurized with an IOP applied to the posterior surface of the cornea.
• Second step - Flap cut and ablation: the flap is cut by deactivating the previously defined contact between the flap and the rest of the cornea; afterward, the ablation volume is removed, and a superficial pressure of P = 0.01 mmHg is applied on the flap to push it down and activate the contact between its bottom surface and the post-surgical cornea.
• Third step - Equilibrium step: in this last step, the previously applied pressure P is removed in order to allow the model to reach its equilibrium state.

After the flap was cut, no changes to its material properties (e.g., transitioning from an anisotropic to an isotropic material model (Dupps and Seven, 2016)) were applied. Only the contact at the new interface created during the surgery was defined. For further details on the mechanical influence of the material model on flap behavior, refer to Supplementary Figure S1 in the Supplementary Material.

#### 2.3.3 SMILE

In the SMILE model, a 6.5 mm lenticule was created and placed at a 120 µm of depth with respect to the anterior surface, constituting the cap thickness, as it is done in clinical practice (Lv et al., 2023). The cap diameter is usually set as 7.6 mm and determines the dimension of the pocket that is created to extract the lenticule. When performing SMILE in daily clinical practice, a 10% is added to the target correction to ensure optical accuracy and an additional 15 µm thickness is added to the lenticule to avoid rupture during its surgical extraction (Wang et al., 2022). Therefore, a correction of −4.4 D was set in order to correct −4 D with a total lenticule thickness of 85 μm, to replicate surgical conditions. The RSB, considering cap and lenticule thickness, was of 374 µm.

SMILE surgery consisted of two steps (Figure 3):

• First step - Pressurization: the model is pressurized with an IOP applied to the posterior surface of the cornea.
• Second step - Lenticule extraction: the lenticule is removed and a hard self-contact with friction coefficient of 0.5 is defined to avoid penetration of the two internal corneal surfaces, that come into contact due to the action of the IOP.

As in the LASIK model, no changes to the cap material properties were applied in the SMILE model (see Supplementary Material for further details).

### 2.4 Pre- and post-surgical optomechanical analyses

In the following section, the analyses conducted to investigate the onset and progression of ectasia in both pre- and post-surgical conditions will be presented.

In the first analysis, the optomechanical response of the constitutive model to a localized circular area of corneal tissue undergoing degradation was investigated, to assess its ability to reproduce ectasia progression, characterized by corneal bulging and thinning. For this analysis, the material properties of the model ( $C_{10}$ , $k_{1}$ , $k_{ip}$ and $k_{op}$ ) were degraded at once, as it is likely that more processes coexist during the development of the disease.

In the second analysis, the effect of three laser surgeries on the post-surgical corneal model was examined in both healthy and pathological scenarios, aiming to identify which procedure induces the greatest optomechanical changes in the presence of a softened area. As for the previous analysis, the material properties ( $C_{10}$ , $k_{1}$ , $k_{ip}$ and $k_{op}$ ) were degraded at once.

In all simulations performed in this study, the zero-pressure algorithm was first applied to the healthy cornea, after which the pathological tissue was introduced.

To evaluate ectasia progression across increasing severity stages, both mechanical and optical analyses were conducted in pre- and post-surgical configurations. From a mechanical perspective, the stress and strain distributions within the model were examined. On the optical side, the Belin ABCD staging system (Belin et al., 2020) was used, as it is the classification currently employed in clinical practice and integrated into the Pentacam topographer by Oculus Optikgeräte GmbH (Wetzlar, Germany).

This staging system assigns a severity grade to corneas that developed ectasia, based on four parameters (ABCD): A is the anterior surface curvature in a 3 mm zone centered on the thinnest pachymetry (K_m AS_ in Table 3); B is the posterior surface curvature in a 3 mm zone centered on the thinnest pachymetry (K_m PS_); C is the thinnest pachymetry point in µm and D is distance best corrected visual acuity. Depending on the computed values, five different stages for classifying keratoconus can be assigned (see (Belin et al., 2020) for more details). In this work, an algorithm that computed the parameters (ABC) was developed. Parameters A and B were computed as the tangential curvature of the center of the cone at the anterior and posterior surfaces respectively (the center of the cone is known, since it is the center of the degraded zone), by performing a sphere fitting at each point of the anterior surface (Doll et al., 2020). Tangential curvature is measured in diopters (D), derived by multiplying its value with the difference in refractive indices between two media of interest (Klein and Mandell, 1995) (Equation 16):

$$K_{m}=\frac{n_{2}-n_{1}}{R}$$ (16)

For the anterior surface, $n_{2}=1.3375$ represents the keratometric index 1.3375 and $n_{1}=1$ corresponds to the refractive index of the air. For the posterior surface $n_{2}=1.336$ represents the refractive index of the aqueous humor and $n_{1}=1.376$ corresponds to the refractive index of the cornea. These values align with the refractive indices used by the Pentacam topographer to compute standard keratometric values. Parameter C was calculated as the minimum normal distance between the anterior and posterior surface nodes. Parameter D could not be included in the study as it is not machine generated and depends on the patient’s subjective visual acuity.

**TABLE 3:** Belin classification for the heterogeneous model at increasing pathology severity.

γ	K_m AS_ [D]	K_m PS_ [D]	Thinnest pachymetry [µm]	Belin ABCD
1	40.90	−5.95	578.84	A0, B0, C0
0.9	41.61	−6.05	574.94	A0, B0, C0
0.8	42.61	−6.18	570.18	A0, B0, C0
0.7	43.91	−6.36	564.35	A0, B0, C0
0.6	45.63	−6.59	556.98	A0, B0, C0
0.5	47.94	−6.89	547.22	A1, B1, C0
0.4	51.66	−7.39	531.83	A2, B2, C0
0.3	56.03	−7.96	514.31	A4, B3, C0
0.2	62.64	−8.77	488.82	A4, B4, C1
0.1	68.18	−9.38	463.47	A4, B4, C1

## 3 Results

In the following sections, the main results of the conducted analysis are presented. First, the impact of the degradation of the tissue components on the model is analyzed in a pre-surgical scenario. Then, the effect of the different refractive procedures on the final optomechanical outcome at both healthy and pathological conditions is investigated.

### 3.1 Pre-surgical evaluation of corneal tissue degradation

In this first analysis, ectasia progression in the model was evaluated relative to healthy homeostatic conditions (i.e., before performing the surgery, when the model is pressurized only), by introducing a weakened area with progressively reduced stiffness.

**FIGURE 4:**
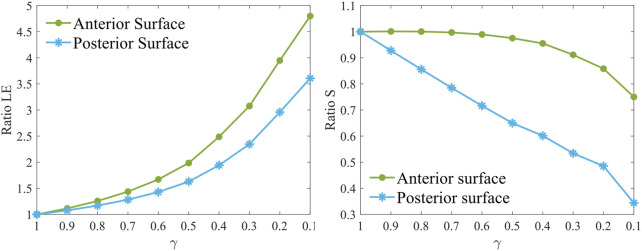
Maximum principal strain ratio (left) and maximum principal stress ratio (right) for increasing pathology severity (γ = 1.0–0.1) in the degraded area at the anterior (AS) and posterior (PS) surfaces at pre-surgical homeostatic conditions. The ratio is computed with respect to pre-surgical healthy conditions (γ = 1).

From a mechanical perspective, the evolution of the principal maximum strain and stress was evaluated by computing their ratio relative to the healthy case (γ = 1). The ratio for each stiffness reduction was computed relative to pre-surgical healthy conditions (γ = 1) by averaging the stress and strain values of five elements selected from the pathological area at both the anterior and posterior surfaces, respectively. As the pathology worsens, the strains on the anterior and posterior surfaces of the weakened area increase non-linearly (Figure 4), increasing up to 5 times the healthy value on the anterior surface and up to 3.5 times on the posterior surface in the most severe case. In contrast, stresses at the anterior surface remain almost constant until a 60% loss of material properties has occurred (γ = 0.4), after which they decrease rapidly to 80% relative to healthy conditions. On the posterior surface, the maximum principal stress ratio follows a linear downward trend, ultimately decreasing by 70%.

**FIGURE 5:**
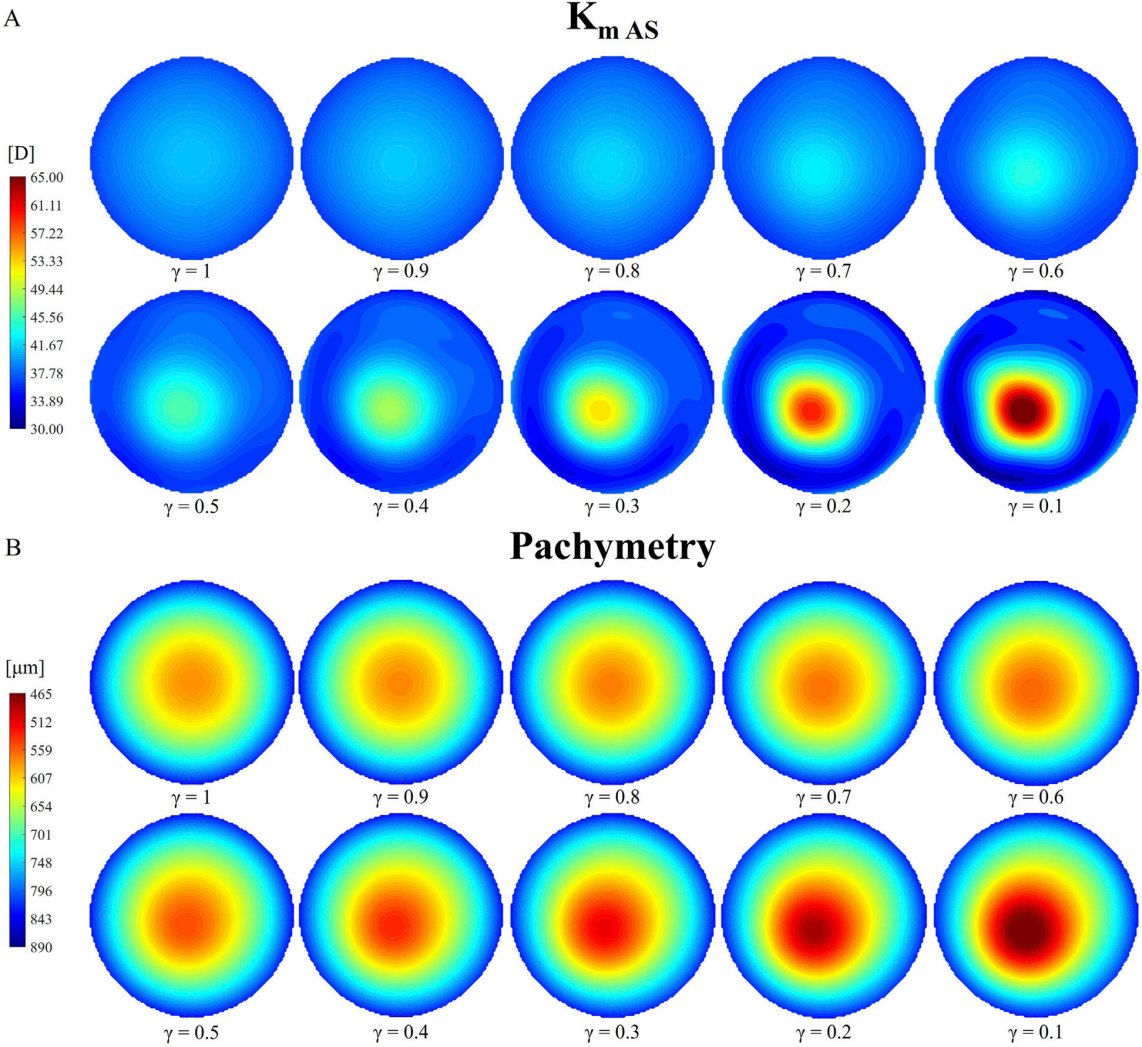
Evolution of tangential curvature of the anterior surface [K_m AS_, **(A)**] and pachymetry thinning (B) with increasing pathology severity (γ = 1.0–0.1) under pre-surgical homeostatic conditions.

From an optical perspective, the Belin ABCD staging system was used to assess ectasia progression and evaluate the model’s ability to replicate varying degrees of disease severity (Table 3; Figure 5).

Table 3 shows that the anterior and posterior curvatures (K_m AS_ and K_m PS_) gradually increase starting from the less severe cases, while pachymetry decreases. The anterior curvature increases slowly in the degraded area, until a 50% loss of mechanical properties is reached. Beyond this point, the progression of the cone accelerates, resulting in a final curvature increase of approximately 28 D with respect to healthy conditions (γ = 1). A similar behavior is observed on the posterior surface, although the increase in curvature is smaller compared to the anterior surface (approximately −3 D). When the material properties are reduced by 50% (γ = 0.5), the cone reaches stage 1 severity based on the anterior and posterior surface curvatures (A1 and B1, Table 3). Beyond this point, ectasia progresses rapidly, until reaching stage 4, the most severe classification according to the Belin classification system in terms of curvature values (A4 and B4). Both the anterior and posterior surfaces presented similar percentage changes in dioptric changes from the healthy state to the most severe condition.

Figure 5 presents a qualitative representation of the information reported in Table 3, showing the tangential curvature maps of the anterior surface and the pachymetry for each reduction in stiffness γ considered. The tangential curvature maps of the posterior surface are omitted to avoid redundancy. An asymmetry in the curvature map is already visible when a 30% loss of mechanical properties has occurred (γ = 0.7), where the location of the pathological zone becomes noticeable.

The pachymetry map is less sensitive to micrometric node displacements compared to the tangential curvature. Therefore, thinning in the model becomes more noticeable from cases with γ = 0.6–0.5. As the pathology progresses, both the points of highest curvature and thinnest pachymetry shift toward the center of the degraded area, which also expands in size. Similar to the evolution of anterior and posterior tangential curvature, pachymetry decreases more gradually in cases of less severe stiffness loss (approximately 30 microns for γ = 0.9–0.5). As the pathology worsens, the thinning progresses more rapidly. The model exhibits a final pachymetry decrease of 115.4 microns relative to the initial central corneal thickness (CCT), classified as a stage 1 cone (C1).

### 3.2 Analysis of the post-surgical impact of laser treatments on the pathological cornea

**FIGURE 6:**
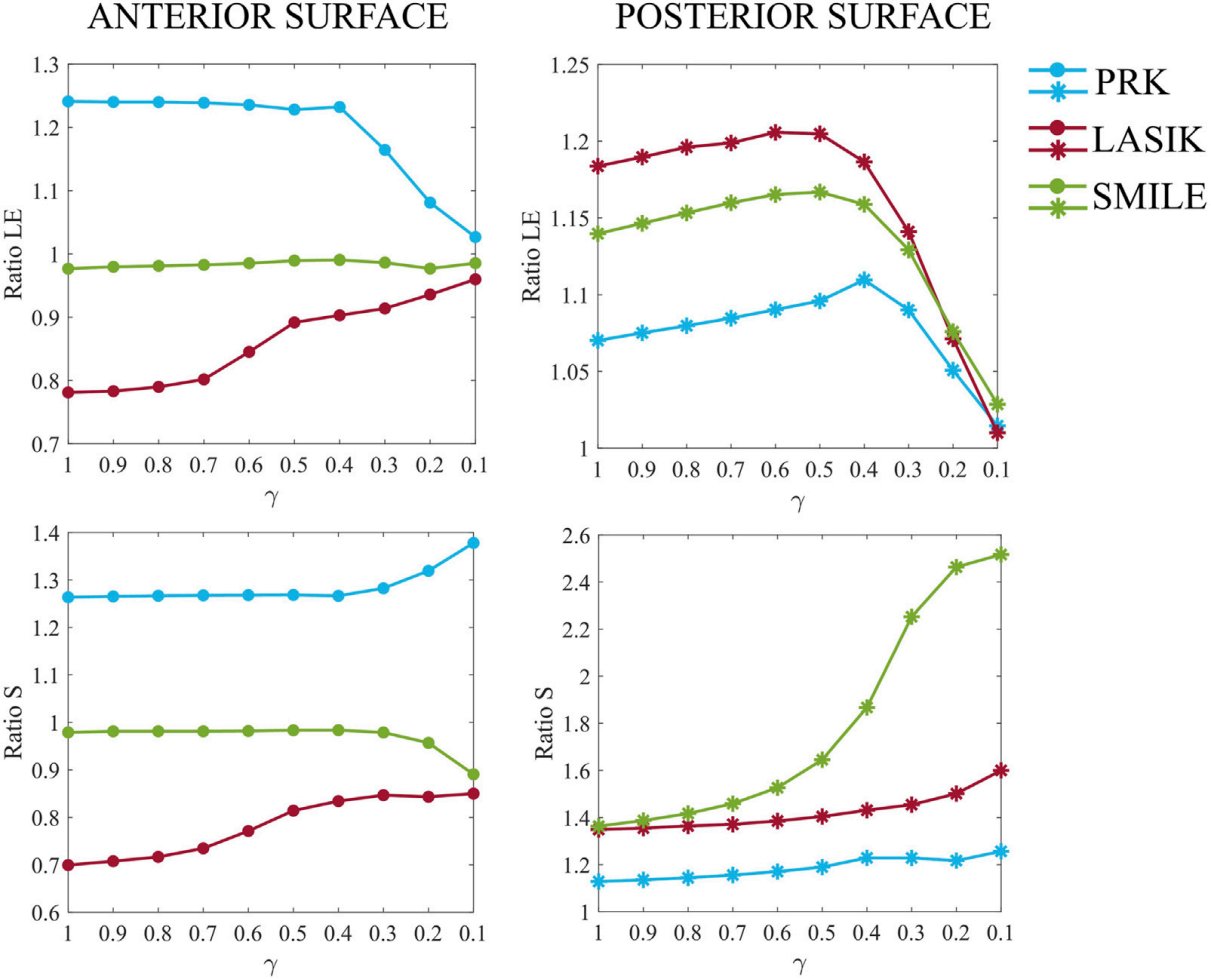
Maximum principal logarithmic strain and maximum principal stress ratios relative to pre-surgical conditions in the degraded area at the anterior (left) and posterior (right) surfaces, for increasing pathology severity (γ = 1.0–0.1).

In this second analysis, the optomechanical impact of three refractive procedures (PRK, LASIK and SMILE) on the pathological cornea was investigated. Figure 6 shows the ratios of the maximum principal stress and the maximum principal logarithmic strain in the degraded area on the anterior and posterior surfaces, for increasing pathology severity (γ = 1.0–0.1). Stress and strain values of the 10 elements previously selected in the pathological area (five elements at the anterior surface and five at the posterior) were averaged. The ratios were calculated relative to the pre-surgical condition of each specific pathological case to isolate changes caused solely by the refractive procedures (e.g., *Ratio S = S*_*Post-surgical (γ=0.1)*_ */S*_*Pre-surgical (γ=0.1)*_ *)*.

Analyzing Figure 6 in detail, differences among the three procedures are evident even in the healthy case (γ = 1.0). PRK surgery caused an approximate 25% increase in both strains and stresses on the anterior surface, whereas its effect on the posterior surface was less pronounced, resulting in a 7% increase in strain and a 13% increase in stress. These increments remained constant on the anterior surface until a 60% loss of material properties (γ = 0.4) occurred, suggesting that the surgery did not exacerbate the pre-existing pathological condition of the cornea. Beyond this point, strains decrease toward a ratio of one due to the fact that the model is already highly deformed in pre-surgical conditions, and the surgery no longer induces additional deformation. In contrast, stresses increase by 10% compared to less severe cases. At the posterior surface, strains increase linearly until γ = 0.4, after which the ratio decreases toward 1, similar to the behavior observed on the anterior surface. Stresses increase by up to 10% compared to healthy conditions (γ = 1.0), as observed on the anterior surface.

LASIK resulted in a decrease in both strains and stresses on the anterior surface (22% and 30%, respectively) in the healthy case (γ = 1.0), while causing a greater increase in both strains and stresses on the posterior surface (18% and 35%, respectively) compared to PRK. When the degraded area is introduced and the pathology progresses (γ = 0.9–0.1), the stress and strain ratios on the anterior surface increase non-linearly towards 1, indicating that surgery does not worsen the severity of the disease, similar to what was observed for PRK. On the posterior surface, a similar evolution to PRK is observed for both strain and stress ratios, but shifted by 10% and 22%, respectively, due to the higher mechanical impact of LASIK on the posterior cornea. Compared to γ = 1, stresses increased by 25% on the posterior surface in the most severe case (γ = 0.1).

Regarding the SMILE procedure, the strain ratio on the anterior surface remains consistently close to one across all stiffness reduction levels γ considered. The stress ratio, on the other hand, remains constant up to γ = 0.4, after which it decreases to a value close to 9%. For the posterior surface, SMILE exhibits a similar trend in strain ratio to that observed in PRK and LASIK, with an initial increase of 14% compared to pre-surgical healthy conditions. This positions SMILE at an intermediate value among the three procedures. The strain ratio increases gradually until γ = 0.4, after which it decreases toward a ratio of 1. In terms of stress, SMILE causes a 36% increase in post-surgical stresses on the posterior surface under healthy conditions, similar to what is observed with LASIK. However, as the pathology progresses, SMILE displays a distinct behavior compared to the other procedures. The stress ratio follows a sigmoid trend, ultimately reaching a final value 2.5 times higher than its corresponding pre-surgical pathological configuration.

More generally, LASIK caused a decrease in both stresses and strains on the anterior surface, whereas PRK led to an increase. On the contrary, SMILE had little to no effect on stresses and strains on the anterior surface. On the posterior surface, all three procedures caused an initial increase in both stress and strain values. The greatest deformation on the anterior surface was observed for the PRK procedure, whereas the posterior surface was most deformed by LASIK. In terms of the stresses, PRK caused the highest increase on the anterior surface, whereas LASIK and SMILE similarly affected the stresses on the posterior surface, resulting in an approximate 35% increase under healthy conditions (γ = 1.0).

**FIGURE 7:**
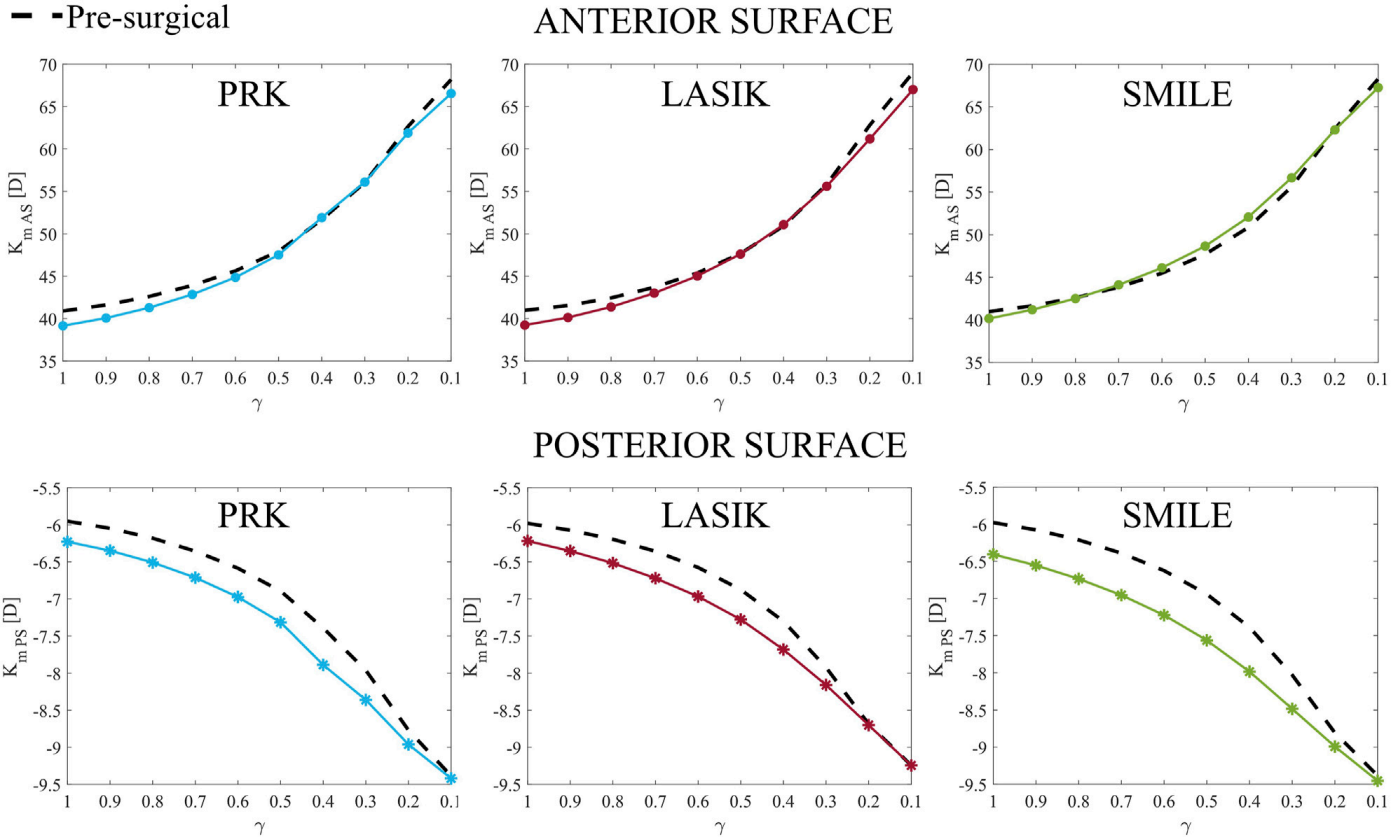
Anterior (K_m AS_, top) and posterior (K_m PS_, bottom) post-surgical curvature evolution at increasing disease severity (γ = 1.0–0.1) in the pathological area. They represent parameters A and B of Belin grading system.

Moving to the optical analysis, we compared anterior and posterior post-surgical curvatures in the pathological area with respect to the pre-surgical condition (Figure 7). In a healthy cornea, when laser refractive surgery is performed, the post-surgical curvature of the anterior surface decreases, whereas the curvature of the posterior surface increases due to the removal of ablation tissue (see Figure 7, γ = 1). This effect is more pronounced on the posterior surface due to the direct action of intraocular pressure (IOP).

As the material degrades, the curvature of both the anterior and posterior surfaces increases, with the difference in curvature between pre-surgical and post-surgical conditions decreasing as degradation progresses. On the anterior surface, this difference becomes almost negligible for γ < 0.5 in all cases. By contrast, on the posterior surface, the post-surgical curvature is always greater than the pre-surgical curvature, becoming equal only when γ close to 0.1.

## 4 Discussion

Despite the widespread use of laser refractive surgeries in clinical practice, their potential role in keratoconus development remains highly debated (Moshirfar et al., 2021). This study aimed to analyze the optomechanical impact of three laser refractive procedures (PRK, LASIK, and SMILE) on both healthy and pathological corneas at varying stages of disease severity. A finite element analysis was conducted to determine whether any procedure could significantly exacerbate the pathology, highlighting its potential role in ectasia progression. Through FE models, we were able to investigate the effects of refractive procedures in pathological cases that would not typically be treated in clinical practice. In fact, when a patient is suspected to be at risk of post-surgical ectasia during pre-surgical screening, surgeons opt not to proceed with the surgery. Since it remains unclear whether keratoconus primarily develops as a post-surgical complication, i.e., as an iatrogenic disease, or if surgery exacerbates a pre-existing condition, as discussed by (Moshirfar et al., 2021), the relationship between refractive procedures and keratoconus progression was investigated.

In the first analysis of this work (Section 3.1), ectasia progression was evaluated at homeostatic conditions, by introducing a weakened area with progressively reduced stiffness. Most changes occur when the model is pressurized, as the stress-free configuration was computed only for the healthy case (γ = 1), and the pathological area was introduced afterward. The unpressurized geometry was computed only for the healthy case to analyze when the cone might develop following the onset of a weakened area. Introducing the pathological area before computing the zero-pressure configuration would prevent the cone from forming when the model is returned to its homeostatic condition, even in the presence of a softer pathological area, which would lack physical relevance.

As the pathology progresses, strains increase non-linearly, while stresses decrease following a more linear trend (Figure 4). Strain plays a key role in regulating cell activation to maintain and remodel the ECM under healthy homeostatic conditions. The pivotal role of strain as a driving force for ectasia progression was also discussed in a previous study by our group (Fantaci et al., 2024b). It has been hypothesized that alterations in protein expression observed in keratoconic corneas may cause oxidative damage, leading to ECM degradation (Santodomingo-Rubido et al., 2022; Kenney and Brown, 2003) and changes in the strain distribution within corneal tissue. As a result of ECM degradation, it loses its adhesive function, causing collagen fibers to shift, eventually break, and lose their specific orientation. The major mechanical changes occurring on the posterior surface may indicate that the structural changes due to tissue degradation observed in pathological corneas could initiate from the posterior surface and propagate towards the anterior, as previously hypothesized by various authors (Akhtar et al., 2008; Alkanaan et al., 2017; Falgayrettes et al., 2023).

To evaluate the progression of the disease from an optical perspective, the Belin ABCD staging system was used. As shown in Table 3, the disease begins to progress already from the less severe cases. Once a 50% loss of tissue stiffness is reached, the pathology worsens more rapidly, reaching stage 4 in the most severe cases (γ = 0.2–0.1) for both anterior and posterior curvatures (A4 and B4) in the area where the cone has developed. Tangential curvature is more sensitive to micrometric node displacements and better highlights geometric changes in the model compared to pachymetry maps (Figure 5). In fact, curvature maps allowed us to identify cone development from the earliest stages of the disease.

Pachymetry thinning, along with localized curvature steepening, is a characteristic sign of ectasia development. Our model showed a final pachymetric thinning of 115 microns compared to the initial healthy CCT (578.84 microns) and was classified as stage 1 (C1, Table 3) according to the Belin classification. This result highlights a limitation of Belin ABCD staging system. Despite the model showing a significant reduction in pachymetry (115.4 microns), the initial CCT in the model was higher than the average (usually around 550 microns), leading to the most severe case being classified as only grade 1. This is because Belin system does not account for the relative change in pachymetry from its initial value. Moreover, tracking disease progression is challenging, as patients need to undergo check-ups before the disease develops and be monitored annually. This is likely why the Belin staging system does not consider parameter evolution over time.

When the initial pachymetry is higher than average (as in this case), it becomes more challenging for the model to reach lower pachymetric values as the mechanical properties deteriorate. If the model’s initial CCT had been lower (490–550 microns), as is common in many patients, a final pachymetric thinning of 115.4 microns would have corresponded to stage 2 or three according to the Belin classification. However, the constitutive model used in this study (Equation (1)) captures the thinning behavior only up to a certain extent. A maximum thinning of 115.4 microns (Table 3) is achieved for the highest stiffness loss (γ = 0.1). Beyond this point, the model cannot reproduce further cone progression, as numerical issues would arise when tissue stiffness is further reduced. This result may suggest that a purely elastic continuum-based formulation may not be appropriate to mimic advanced stages of the disease (stage 4) in terms of pachymetry thinning, as it is not able to capture the complex processes of structural disorganization occurring in the stroma during disease’s progression, as highlighted in our previous work (Fantaci et al., 2024b).

In the second analysis of this work, the optomechanical impact of three refractive procedures on healthy and pathological corneas was investigated. PRK surgery had the highest mechanical impact on the anterior surface and the lowest on the posterior. LASIK showed an opposite behavior with respect to PRK, as it lowered both stresses and strains on the anterior surface, while it had a higher impact on the same variables on the posterior surface. LASIK significantly affects the posterior surface because the creation of the flap (or the cap in SMILE) during the procedure introduces a geometrical discontinuity in the corneal thickness, leaving the anterior surface nearly unloaded. As pointed out in (Montanino et al., 2023), while cap in SMILE partially collaborates in sustaining the IOP, flap contribution in LASIK to the IOP is almost null. Consequently, stresses and strains concentrate on the posterior cornea, which is further exacerbated by a thinner RSB in LASIK (419 microns) compared to PRK (519 microns). This may be attributed to the limited healing capacity of corneal tissue after being cut, with only 2.4% of its original strength being recovered (Schmack et al., 2005).

For the healthy case (γ = 1), SMILE showed an intermediate mechanical impact, falling between PRK and LASIK, as also observed in (Montanino et al., 2023). As the disease progresses to more severe stages, SMILE was found to be the treatment that caused the highest increase in stresses on the posterior surface (Figure 6). This consistent increase in the stresses is probably due to the fact that a thicker lenticule is removed in this surgical procedure to avoid its rupture during the extraction, leaving a lower percentage of RSB (374 microns). In the model, the posterior portion of the cornea is around a 50% softer (Randleman et al., 2008; Reinstein et al., 2013) with respect to the anterior part. Having a lower percentage of RSB at the softer posterior cornea lead to higher stresses that may promote the progression of the pathology. This would also explain why the risk of post-surgical ectasia is higher when the RSB is lower than 250 microns and the surgeons prefer not to operate (Kim et al., 2007). In general, it is recommended to preserve the RSB as much as possible to minimize the risk of post-surgical complications. In this study, the RSB remained above the threshold in all three simulated surgical procedures.

Moreover, careful consideration should be given when selecting the appropriate treatment for each patient, taking into account which procedure induces the highest deformations, particularly on the posterior surface, where ectasia is believed to originate (Falgayrettes et al., 2023; Fantaci et al., 2024b). Currently, the mechanical impact of different laser refractive surgeries is often overlooked in clinical practice, and FE models could help bridge this gap.

In this work, the introduction of pathological tissue into the model did not amplify the mechanical impact of the refractive treatments: stresses and strains remained constant in the majority of the cases until a 60%–70% loss of mechanical properties occurred, suggesting that the refractive procedures were not worsening the pre-existing pathological condition, except for SMILE, that caused larger stresses on the posterior surface for more severe pathological cases. As the pathology progressed (γ = 0.4–0.1), the changes in the strain field with respect to the pre-surgical situation are very small, since the cornea was already highly deformed at pre-surgical conditions due to tissue degradation and the procedures were not causing any further deformation.

From an optical perspective, as the severity of the pathology worsened, none of the three procedures appeared to exacerbate the cone’s development in terms of anterior and posterior curvature (Figure 7) compared to pre-surgical conditions (dashed line), as they demonstrated similar behavior. This observation suggests that surgeries may play a limited role in directly causing post-surgical ectasia. Instead, it seems more plausible that the mechanical changes induced by refractive procedures may accelerate the progression of an underlying pathological condition rather than being the primary cause of an iatrogenic disease. However, long-term observation was not included in this study to analyze the progression of ectasia. As it will be discussed in the study’s limitations, only snapshots of the pathology at progressive severity were analyzed. Our hypothesis for the long-term evolution of the disease is that refractive treatments causing significant mechanical changes in the corneal structure (particularly in the posterior region) carry a higher risk of causing further worsening of the pathology.

In the review by Moshirfar et al. (Moshirfar et al., 2021), LASIK was identified as the surgery with the highest rate of post-surgical ectasia compared to PRK and SMILE. However, this result is influenced by the fact that LASIK is the most commonly performed procedure, inherently increasing the likelihood of encountering post-surgical complications. Although SMILE was developed to be less invasive and more preserving of stromal tissue, it remains relatively new, making it challenging to determine definitively whether it is safer than LASIK. While SMILE avoids the creation of a flap, the removal of a lenticule from the corneal thickness introduces a geometric discontinuity, likely resulting in the residual load being entirely borne by the RSB rather than the cap above the lenticule removal site. In our study, SMILE was found to exert the greatest mechanical impact on the posterior region of pathological corneas in the immediate post-surgical scenario. This finding suggests that SMILE could potentially increase the risk of ectasia progression in a pathological cornea over the long-term. PRK, on the other hand, shows a lower rate of post-surgical ectasia compared to LASIK (Moshirfar et al., 2021), likely because it involves only surface ablation, thereby preserving a larger percentage of the RSB. In this study, we observed that the posterior cornea was minimally affected by PRK, even in the most severe cases, making it less likely to promote the progression of ectasia, when present.

In summary, the three refractive procedures exhibited distinct mechanical behaviors in both healthy and pathological corneal models. However, their impact on the final cone severity, as classified by the Belin system, was equivalent in the immediate post-surgical scenario. To ensure safety, the refractive treatment with the lowest mechanical impact should be selected, as it may reduce the long-term risk of ectasia progression.

Finally, this work is not exempt from limitations that may restrict the observations drawn from this study. First, time has not been considered as variable to evaluate the progression of the disease; specific moments of the disease were analyzed, but the disease progression over time could not be evaluated: in this way, the effect of the surgeries on the pathological cornea is evaluated in the immediate post-surgical condition and not after some time (months or years), which can be relevant in ectatic diseases. In the future, time should be included in the simulations to analyze the post-surgical impact of the refractive procedure in the long-term.

As already discussed above, the choice of a continuum-based purely elastic formulation may not be the most appropriate for mimicking advanced stages of ectasia disease, as it appears to be incapable of capturing the complex processes undergoing at the microscopic level in corneal tissue, that cause a significant reduction of the thickness. A growth model better replicates advanced stages of ectasia, as it is able to capture the structural changes associated with the disease (Fantaci et al., 2024b).

Patient-specific data were not included in this study, as the aim was to eliminate inter-patient variability due to the specific geometrical characteristics of individual corneas. In future work, it would be valuable to investigate the impact of refractive procedures on patients who developed post-surgical ectasia, acknowledging the challenges in acquiring such data. With the versatility of FE models, it may be possible to assess whether a specific surgical procedure would have a greater or lesser impact on patient’s corneas. This approach could also serve as a decision-making tool for surgeons, aiding in the selection of the most appropriate procedure for individual patients.

## 5 Conclusion

This study presented an optomechanical analysis of healthy and pathological corneas before and after laser refractive surgery. The analysis focused on the immediate post-surgical response of corneal tissue to different refractive procedures and demonstrated that, even in the most severe cases, the surgeries did not exacerbate the pre-existing pathological condition. These findings suggest that laser refractive surgeries might act as a catalyst for the progression of a sub-clinical pathological state of the corneal tissue rather than being the primary cause of post-surgical ectasia in entirely healthy corneas. Among the three procedures, PRK was found to be the least invasive treatment from a mechanical perspective, making it less likely to promote the progression of ectasia, when present. In contrast, SMILE showed the greatest impact on the posterior surface in more advanced stages of the disease, suggesting a potential long-term risk for ectasia progression.

## Data Availability

The original contributions presented in the study are included in the article/Supplementary Material. Further inquiries can be directed to the corresponding author.

## Supplementary material

SUPPLEMENTARY FIGURE S1 Distribution of maximum principal stress and maximum principal logarithmic strain in the LASIK model, with anisotropic (left) and isotropic (right) material models assigned to the post-surgical flap. Very small differences in stress and strain distribution are observed when varying the flap material properties in the LASIK model, suggesting they can be neglected.
