# Supplementary figures and images for: Unraveling the impact of laser refractive surgery on corneal ectasia: an in silico study
Source: Front Bioeng Biotechnol. 2025 Feb 26;13:1548539. doi: 10.3389/fbioe.2025.1548539 (PMC11897516; doi:10.3389/fbioe.2025.1548539)

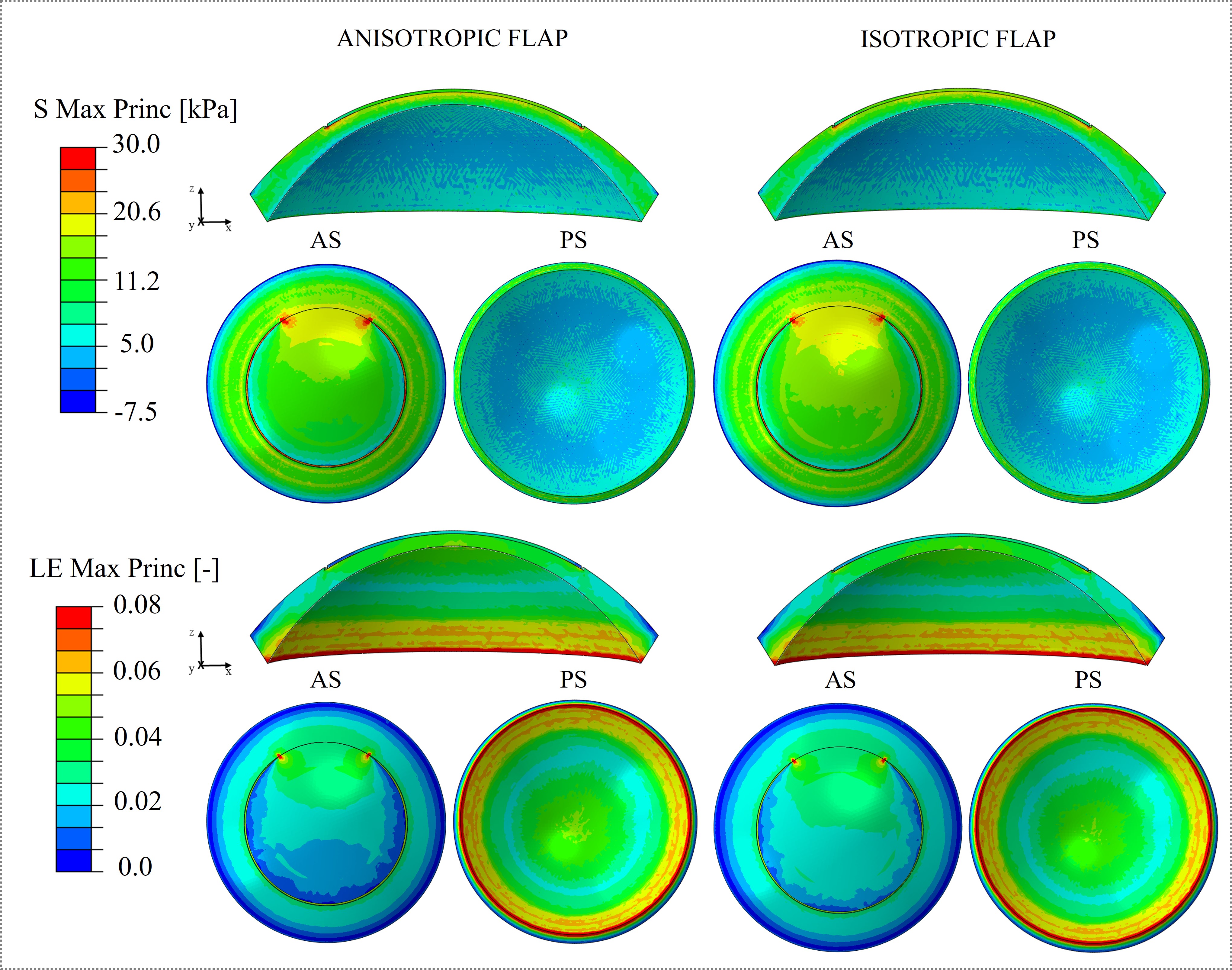

Supplement: Supplementary file 1 [file Image1.jpeg]
